# Supplementary material for: Absence of localization in interacting spin chains with a discrete symmetry
Source: Nat Commun. 2023 Jun 24;14:3778. doi: 10.1038/s41467-023-39468-4 (PMC10290695; doi:10.1038/s41467-023-39468-4)
Supplement: Supplementary file 1 — Supplementary Information [file 41467_2023_39468_MOESM1_ESM.pdf]

Supplementary Information: Absence of localization in interacting spin chains with a discrete symmetry

Benedikt Kloss,[1] Jad C. Halimeh,[2,3] Achilleas Lazarides,[4] and Yevgeny Bar Lev[5]

[1] *Center for Computational Quantum Physics, Flatiron Institute, 162 Fifth Ave, New York, NY 10010, USA*
[2] *Department of Physics and Arnold Sommerfeld Center for Theoretical Physics (ASC), Ludwig-Maximilians-Universität München, Theresienstraße 37, D-80333 München, Germany*
[3] *Munich Center for Quantum Science and Technology (MCQST), Schellingstraße 4, D-80799 München, Germany*
[4] *Interdisciplinary Centre for Mathematical Modelling and Department of Mathematical Sciences, Loughborough University, Loughborough, Leicestershire LE11 3TU, UK*
[5] *Department of Physics, Ben-Gurion University of the Negev, Beer-Sheva 84105, Israel*

May 31, 2023

LIST OF FIGURES

S1 **Excitation profile**. Infinite-temperature, infinite time average of connected spin-spin correlation function $\overline{G^{\infty}_{i,j}}$, for Stark-MBL system with $\gamma = 2.75$ (left panels) and symmetrized-MBL system with $W = 9$ (right panels) in the zero-magnetization sector. Top panels show autocorrelator for $j = \lceil L/2 \rceil$ as function of L on log-log scale, while bottom panels show the spatial profile for several system sizes (darker shades indicate larger systems). 7

S2 **Spreading of the excitation with time**. Dynamics of the (positive) mean-squared displacement for Stark-MBL system with $\gamma = 2.75$ (left panel) and symmetrized-MBL system with $W = 9$ (right panel), plotted on semilog scale. The darker shade corresponds to zero-magnetization, while the lighter shade indicates results from a finite-magnetization sector. The standard deviation over disorder realizations for the symmetrized MBL is indicated by shaded areas. 8

S3 ***Memory of initial condition at infinite time.*** *Left panel*: Infinite-time average of the magnetization profile starting from initial states related to the Néel state for the symmetrized-MBL system with $W = 9$ and L=16. Results are shown for the Néel state (dark red), $|\uparrow\downarrow\uparrow\downarrow\uparrow\downarrow\uparrow\downarrow\uparrow\downarrow\uparrow\downarrow\uparrow\downarrow\uparrow\downarrow\rangle$ and inverted Néel state, with the spins on the right half of the lattice flipped, $|\uparrow\downarrow\uparrow\downarrow\uparrow\downarrow\uparrow\downarrow\downarrow\uparrow\downarrow\uparrow\downarrow\uparrow\downarrow\uparrow\rangle$. *Right panel*: Asymptotic imbalance of the Néel state as a function of system size for even system sizes, plotted on a log-log scale. 8

S4 **Spreading of a single electron**. The electron is initialized at site $x_0 = 5$, in a 1D lattice with mirror-symmetric on-site potential, $\mu_i = \mu_{-i} \in [-8, 8]$ and uniform nearest-neighbour hopping amplitude $t = 1$. The results are obtained from averaging over 1000 disorder realizations per system size. Left panel: Mean-squared displacement of the electronic density $\sigma^2(t) = \sum_{i=1}^{L} (i - x_0)^2 \rho_i(t)$ with as a function of time on semi-log scale. Right panel: Late-time average of the electronic density profile. 9

CONTENTS

List of Figures 2

Supplementary Note 1. Noninteracting systems 3

Supplementary Note 2. Quasi-degeneracies for the Stark-MBL 3

Supplementary Note 3. Infinite time delocalization 4

Supplementary Note 4. Spatial profile of the spin excitation 7

Supplementary Note 5. Excitation spreading 7

Supplementary Note 6. Symmetry breaking of initial conditions 8

Supplementary Note 7. Anderson Localization in Mirror-Symmetric Potential 9

References 9

Supplementary Note 1. NONINTERACTING SYSTEMS

In this section we examine the properties of noninteracting *fermionic* systems, which conserve the total particle number and are symmetric with respect to $\hat{P}$ (see main text). Specifically we consider,

$$\hat{H} = \sum_{i \neq j} f(i-j)\, \hat{c}_i^\dagger \hat{c}_j + \sum_i v_i \left(\hat{n}_i - \frac{1}{2} \right), \tag{S1}$$

where $\hat{c}_i^\dagger$ creates a fermion at site i, $\hat{n}_i = \hat{c}_i^\dagger \hat{c}_i$ is the fermion density operator, $f(i-j)$ corresponds to the hopping rate of the fermions and v_i is the external potential. Due to hermiticity the hopping rate satisfies, $f(i-j) = f^*(j-i)$. To show the requirements on $f(i-j)$ and v_i for the Hamiltonian to be symmetric under $\hat{P}$ it is convenient to use the Jordan-Wigner transformation,

$$\hat{c}_i^\dagger = \left(\prod_{j=1}^{i-1} \hat{\sigma}_j^z \right) \hat{\sigma}_i^+ \qquad \hat{c}_i = \left(\prod_{j=1}^{i-1} \hat{\sigma}_j^z \right) \hat{\sigma}_i^- . \tag{S2}$$

The Hamiltonian written in terms of spins is,

$$\hat{H} = -\sum_{i<j} f(i-j)\, \sigma_i^+ \left(\prod_{k=i+1}^{j-1} \sigma_k^z \right) \sigma_j^- - \sum_{i<j} f^*(i-j)\, \sigma_i^- \left(\prod_{k=i+1}^{j-1} \sigma_k^z \right) \sigma_j^+ + \frac{1}{2} \sum_i v_i \sigma_i^z . \tag{S3}$$

We see that for,

$$\begin{aligned} f^*(i-j) &= f(i-j) \\ f(i-j) &= 0 \qquad |i-j| \mod 2 = 1 \\ v_i &= -v_{\tilde{i}}, \end{aligned} \tag{S4}$$

the Hamiltonian is symmetric with respect to $\hat{P}$. Using Supplementary Eq. (S4) it is easy to check that the corresponding single-particle Hamiltonian,

$$h_{ij} = f(i-j)(1-\delta_{ij}) + v_i \delta_{ij}, \tag{S5}$$

is anti-symmetric with respect to the unitary transformation, $|i\rangle \to (-1)^i |\tilde{i}\rangle$, which means that the single-particle spectrum is symmetric around zero. It is important to note that since the single-particle spectrum is symmetric, there are no exact resonances in the single-body problem, and therefore creating an excitation at site j does *not* create a resonant excitation at the mirrored site $\tilde{j}$, as can be verified numerically. Nevertheless, the many-body spectrum is degenerate, and therefore Assumption 1 is not satisfied and the proof we present in Supplementary Note 3 doesn't apply. Specifically, this includes the noninteracting Stark and the Anderson problem with the anti-symmetric disorder. We have verified numerically that these problems indeed remain localized.

Supplementary Note 2. QUASI-DEGENERACIES FOR THE STARK-MBL

The existence of the quasi-degeneracies can be analytically motivated for the Stark-MBL Hamiltonian,

$$\hat{H} = \sum_{n=1}^{L-1} \left[\frac{J}{2} \left(\hat{S}_n^+ \hat{S}_{n+1}^- + \hat{S}_n^- \hat{S}_{n+1}^+ \right) + \Delta \hat{S}_n^z \hat{S}_{n+1}^z \right] + \sum_{n=1}^{L} \gamma n \hat{S}_n^z . \tag{S6}$$

We define,

$$\hat{H}_0 = \Delta \sum_{n=1}^{L-1} \hat{S}_n^z \hat{S}_{n+1}^z + \sum_{n=1}^{L} \gamma n \hat{S}_n^z , \tag{S7}$$

which is diagonal at the computational basis, namely the eigenbasis of $\hat{S}_n^z$ operators. We will treat the flip-flop term as a perturbation,

$$\hat{V} = \frac{J}{2} \sum_{n=1}^{L-1} \left(\hat{S}_n^+ \hat{S}_{n+1}^- + \hat{S}_n^- \hat{S}_{n+1}^+ \right) . \tag{S8}$$

Our goal is to show that at zero magnetization there are exponentially many states which are almost generate. We call a unit-dipole a configuration which looks like, $d^{+}_{i,i+1} = (\uparrow, \downarrow)$ or equivalently $d^{-}_{i,i+1} = (\downarrow, \uparrow)$. We construct a state $|\psi_1\rangle$ by adding to the lattice N_+ dipoles d^+ and $N_- = L/2 - N_+$ dipoles . The total dipole moment of the state is proportional to, $N_+ - N_-$, and its unperturbed energy is $E^{(0)}_1$. The number of such terms is $\binom{L/2}{N_+}$, namely it is exponential in the size of the system. These states have typically a different energy due to the interaction Δ. By applying the symmetry generator $\hat{P}$ we can obtain a new state $|\psi_2\rangle = \hat{P}\,|\psi_1\rangle$, which has the same unperturbed energy. The operator $\hat{P}$ is a global, while the local perturbation $\hat{V}$ can only flip one unit-dipole at a time. Therefore an order of αL such flips are needed to have a non-vanishing coupling between $|\psi_2\rangle$ and $|\psi_1\rangle$,

$$\langle\psi_1|\,\hat{V}^{\alpha L}\hat{P}\,|\psi_1\rangle \neq 0. \tag{S9}$$

This means that the degeneracy between the eigenvalues is removed only at order αL of the perturbation theory, where α is some constant, which depends on the structure of the state. The resulting splitting between the eigenvalues will be,

$$\delta E_1 \propto J^{\alpha L} = e^{L \ln J}. \tag{S10}$$

Since the typical many-body energy spacing is $\delta = \exp\left[-L\ln 2\right]$, the states will appear quasi-degenerate for,

$$\delta E_1 \ll \delta \qquad J \ll \frac{1}{2}. \tag{S11}$$

Supplementary Note 3. INFINITE TIME DELOCALIZATION

We start by calculation of the correlation function $\left\langle \hat{S}^z_i \hat{S}^z_j \right\rangle_{M=0} = \mathcal{N}_0^{-1}\mathrm{Tr}\left(\hat{P}_0 \hat{S}^z_i \hat{S}^z_j\right)$ for arbitrary spin size. Here $\hat{P}_0$ is a projector on the zero magnetization sector, and $\mathcal{N}_0$ corresponds to the number of states in this sector. Summing over all i we have the sum rule

$$\sum_i \left\langle \hat{S}^z_i \hat{S}^z_j \right\rangle_{M=0} = 0. \tag{S12}$$

Separating the sum to $i = j$ and $i \neq j$ and using the fact that the expectation value cannot depend on either i or j we get,

$$\sum_i \left\langle \hat{S}^z_i \hat{S}^z_j \right\rangle_{M=0} = \left\langle \left(\hat{S}^z_i\right)^2 \right\rangle_{M=0} + (L-1)\left\langle \hat{S}^z_i \hat{S}^z_j \right\rangle_{M=0} = 0. \tag{S13}$$

To proceed we need to calculate $\left\langle \left(\hat{S}^z_i\right)^2 \right\rangle_{M=0}$. Since this is challenging in the zero magnetization sector, we use the asymptotic equivalence of ensembles and calculate $\left\langle \left(\hat{S}^z_i\right)^2 \right\rangle = \mathcal{N}^{-1}\mathrm{Tr}\left(\hat{S}^z_i\right)^2$ with respect to a density matrix proportional to unity, which corresponds to averaging over all magnetization sectors. Since this density matrix is rotationally invariant we have,

$$\left\langle \left(\hat{S}^x_i\right)^2 \right\rangle = \left\langle \left(\hat{S}^y_i\right)^2 \right\rangle = \left\langle \left(\hat{S}^z_i\right)^2 \right\rangle. \tag{S14}$$

Using this and the definition of the magnitude squared operator of the spin

$$\left\langle \left(\hat{S}^x_i\right)^2 + \left(\hat{S}^y_i\right)^2 + \left(\hat{S}^z_i\right)^2 \right\rangle = \left\langle \left(\hat{S}^2_i\right) \right\rangle = s\,(s+1)\,, \tag{S15}$$

where s is the size of the spins, obtain

$$\left\langle \left(\hat{S}^z_i\right)^2 \right\rangle = \frac{s\,(s+1)}{3}. \tag{S16}$$

Therefore in the limit of $L \to \infty$, we have $\left\langle \left(\hat{S}_i^z\right)^2 \right\rangle_{M=0} \to \left\langle \left(\hat{S}_i^z\right)^2 \right\rangle = \frac{s(s+1)}{3}$. For any finite L we will write $\left\langle \left(\hat{S}_i^z\right)^2 \right\rangle_{M=0} = C_s$, where C_s is a constant $O\left(s^2\right)$. For clarity we will drop the $\langle . \rangle_{M=0}$ indication bellow, since all expectations below are taken at zero magnetization. Combining this with the sum-rule Supplementary Eq. (S13) we obtain,

$$\left\langle \hat{S}_i^z \hat{S}_j^z \right\rangle = C_s \begin{cases} -\frac{1}{L-1} & i \neq j \\ 1 & i = j \end{cases}. \tag{S17}$$

The infinite time average of the MSD, which only assumes that there are no exact degeneracies is given by,

$$\overline{\sigma_\infty^2} = \sum_{i=1}^{L} (i-j)^2 \frac{1}{\mathcal{N}} \sum_\alpha \langle\alpha| \hat{S}_i^z |\alpha\rangle \langle\alpha| \hat{S}_j^z |\alpha\rangle - \sum_{i=1}^{L} (i-j)^2 \left\langle \hat{S}_i^z \hat{S}_j^z \right\rangle. \tag{S18}$$

Using Supplementary Eq. (S17) we see that the last term contributes

$$\sum_i (i-j)^2 \left\langle \hat{S}_i^z \hat{S}_j^z \right\rangle = -\frac{C_s}{(L-1)} \sum_i (i-j)^2 = -\frac{C_s}{6} \frac{L\left((L+1)(2L+1) + 6j^2 - 6j(L+1)\right)}{L-1} \sim -\frac{s(s+1)}{9} L^2. \tag{S19}$$

We now move to the first term which can be simplified,

$$\begin{aligned} \frac{1}{\mathcal{N}} \sum_\alpha \sum_{i=1}^{L} (i-j)^2 \langle\alpha| \hat{S}_i^z |\alpha\rangle \langle\alpha| \hat{S}_j^z |\alpha\rangle &= \frac{1}{\mathcal{N}} \sum_\alpha \sum_{i=1}^{L} \left(i^2 + j^2 - 2ij\right) \langle\alpha| \hat{S}_i^z |\alpha\rangle \langle\alpha| \hat{S}_j^z |\alpha\rangle \\ &= \frac{1}{\mathcal{N}} \sum_\alpha \sum_{i=1}^{L} \left(i^2 - 2ij\right) \langle\alpha| \hat{S}_i^z |\alpha\rangle \langle\alpha| \hat{S}_j^z |\alpha\rangle, \end{aligned} \tag{S20}$$

where the last equality follows since we are working a zero magnetization sector. Focusing on, $\sum_{i=1}^{L} i^2 \langle\alpha| \hat{S}_i^z |\alpha\rangle \langle\alpha| \hat{S}_j^z |\alpha\rangle$, and using the parity symmetry

$$\begin{aligned} \sum_{i=1}^{L} i^2 \langle\alpha| \hat{S}_i^z |\alpha\rangle \langle\alpha| \hat{S}_j^z |\alpha\rangle &= \sum_{i=1}^{L} i^2 \langle\alpha| \hat{P}\hat{P}\hat{S}_i^z \hat{P}\hat{P} |\alpha\rangle \langle\alpha| \hat{S}_j^z |\alpha\rangle \\ &= -\sum_{i=1}^{L} i^2 \langle\alpha| \hat{S}_{L-i+1}^z |\alpha\rangle \langle\alpha| \hat{S}_j^z |\alpha\rangle. \end{aligned} \tag{S21}$$

Changing the summation variables, $\tilde{i} = L - i + 1$ gives,

$$\begin{aligned} \sum_{i=1}^{L} i^2 \langle\alpha| \hat{S}_i^z |\alpha\rangle \langle\alpha| \hat{S}_j^z |\alpha\rangle &= -\sum_{i'=1}^{L} \left(L - \tilde{i} + 1\right)^2 \langle\alpha| \hat{S}_{i'}^z |\alpha\rangle \langle\alpha| \hat{S}_j^z |\alpha\rangle \\ &= -\sum_{i=1}^{L} i^2 \langle\alpha| \hat{S}_i^z |\alpha\rangle \langle\alpha| \hat{S}_j^z |\alpha\rangle - (L+1)^2 \sum_{i=1}^{L} \langle\alpha| \hat{S}_i^z |\alpha\rangle \langle\alpha| \hat{S}_j^z |\alpha\rangle \\ &\quad + 2(L+1) \langle\alpha| \overbrace{\sum_i i \hat{S}_i^z}^{\hat{D}} |\alpha\rangle \langle\alpha| \hat{S}_j^z |\alpha\rangle, \end{aligned} \tag{S22}$$

where the second term vanishes at zero magnetization. We can now rearrange the terms to obtain the identity,

$$\sum_{i=1}^{L} i^2 \langle\alpha| \hat{S}_i^z |\alpha\rangle \langle\alpha| \hat{S}_j^z |\alpha\rangle = (L+1) \langle\alpha| \hat{D} |\alpha\rangle \langle\alpha| \hat{S}_j^z |\alpha\rangle. \tag{S23}$$

Inserting this identity into Supplementary Eq. (S20) gives,

$$\frac{1}{\mathcal{N}} \sum_\alpha \sum_{i=1}^{L} (i-j)^2 \langle\alpha| \hat{S}_i^z |\alpha\rangle \langle\alpha| \hat{S}_j^z |\alpha\rangle = (L+1-2j) \frac{1}{\mathcal{N}} \sum_\alpha \langle\alpha| \hat{D} |\alpha\rangle \langle\alpha| \hat{S}_j^z |\alpha\rangle = \left(\tilde{j} - j\right) \frac{1}{\mathcal{N}} \sum_\alpha \langle\alpha| \hat{D} |\alpha\rangle \langle\alpha| \hat{S}_j^z |\alpha\rangle, \tag{S24}$$

where we have defined the reflected $\tilde{j} \equiv L - j + 1$. We note that for zero magnetization L is even and therefore there is no j such that $j = \tilde{j}$. We will now proceed by bounding this term. Using the triangle inequality,

$$\frac{1}{\mathcal{N}} \left| \sum_{\alpha} \langle\alpha| \hat{D} |\alpha\rangle \langle\alpha| \hat{S}_j^z |\alpha\rangle \right| \leq \frac{1}{\mathcal{N}} \sum_{\alpha} \left| \langle\alpha| \hat{D} |\alpha\rangle \right| \left| \langle\alpha| \hat{S}_j^z |\alpha\rangle \right|, \tag{S25}$$

now since,

$$\left| \langle\alpha| \hat{S}_j^z |\alpha\rangle \right| \leq s, \tag{S26}$$

we can bound,

$$\frac{1}{\mathcal{N}} \left| \sum_{\alpha} \langle\alpha| \hat{D} |\alpha\rangle \langle\alpha| \hat{S}_j^z |\alpha\rangle \right| \leq \frac{1}{\mathcal{N}} \sum_{\alpha} \left| \langle\alpha| \hat{D} |\alpha\rangle \right| \left| \langle\alpha| \hat{S}_j^z |\alpha\rangle \right| \leq \frac{s}{\mathcal{N}} \sum_{\alpha} \left| \langle\alpha| \hat{D} |\alpha\rangle \right|. \tag{S27}$$

The right-hand side is not a trace of a matrix, and depends on the basis. However for any diagonalizabe matrix we can change to a basis $|n\rangle$, where $\hat{D}$ is diagonal, such that,

$$\frac{1}{\mathcal{N}} \sum_{\alpha} \left| \langle\alpha| \hat{D} |\alpha\rangle \right| = \frac{1}{\mathcal{N}} \sum_{\alpha} \left| \sum_{n.m} \langle\alpha| n\rangle \langle n| \hat{D} |m\rangle \langle m| \alpha\rangle \right| = \frac{1}{\mathcal{N}} \sum_{\alpha} \left| \sum_{n} d_n \left| \langle\alpha| n\rangle \right|^2 \right|, \tag{S28}$$

using the triangle inequality again we obtain,

$$\frac{1}{\mathcal{N}} \sum_{\alpha} \left| \langle\alpha| \hat{D} |\alpha\rangle \right| = \frac{1}{\mathcal{N}} \sum_{\alpha} \left| \sum_{n} d_n \left| \langle\alpha| n\rangle \right|^2 \right| \leq \frac{1}{\mathcal{N}} \sum_{\alpha} \sum_{n} |d_n| \left| \langle\alpha| n\rangle \right|^2 = \frac{1}{\mathcal{N}} \sum_{n} |d_n|, \tag{S29}$$

which means that $\frac{1}{\mathcal{N}} \sum_{\alpha} \left| \langle\alpha| \hat{D} |\alpha\rangle \right|$ is maximized in the basis where $\hat{D}$ is diagonal. We now use Jensen's inequality and obtain finally,

$$\frac{1}{\mathcal{N}} \sum_{\alpha} \left| \langle\alpha| \hat{D} |\alpha\rangle \right| \leq \frac{1}{\mathcal{N}} \sum_{n} |d_n| \leq \left(\frac{1}{\mathcal{N}} \sum_{n} d_n^2 \right)^{1/2} = \left(\frac{1}{\mathcal{N}} \mathrm{Tr}\, \hat{D}^2 \right)^{1/2} \equiv \left\langle \hat{D}^2 \right\rangle^{1/2}. \tag{S30}$$

The expectation $\left\langle \hat{D}^2 \right\rangle$ can be evaluated exactly using Supplementary Eq. (S17),

$$\begin{aligned} \left\langle \hat{D}^2 \right\rangle &= \sum_{i,j} i\, j \left\langle \hat{S}_i^z \hat{S}_j^z \right\rangle = C_s \left[\left[-\frac{1}{L-1} \right] \left(\sum_{i \neq j} i\, j \right) + \sum_{i} i^2 \right] \\ &= C_s \left[-\frac{\left(\left(\sum_{i=1}^{L} i \right)^2 - \sum_{i=1}^{L} i^2 \right)}{L-1} + \sum_{i=1}^{L} i^2 \right] \\ &= C_s \left[-\frac{L(L+1)(3L+2)}{12} + \frac{L(L+1)(2L+1)}{6} \right] \\ &= \frac{C_s}{12} L^2 (L+1). \end{aligned} \tag{S31}$$

Combining all the results gives,

$$\frac{1}{\mathcal{N}} \left| \sum_{\alpha} \sum_{i=1}^{L} (i-j)^2 \langle\alpha| \hat{S}_i^z |\alpha\rangle \langle\alpha| \hat{S}_j^z |\alpha\rangle \right| \leq s \left| \tilde{j} - j \right| \sqrt{\frac{C_s}{12} L^2 (L+1)} = O\left(L^{3/2} \right). \tag{S32}$$

Comparing to the second-term of the MSD in Supplementary Eq. (S18) we obtain that for $\left| \tilde{j} - j \right| \leq A L^{1/2}$, where $A > 0$ is some constant,

$$\overline{\sigma_\infty^2} \sim \frac{s(s+1)}{9} L^2, \tag{S33}$$

which concludes the proof that at least a fraction of eigenstates in the system are delocalized.

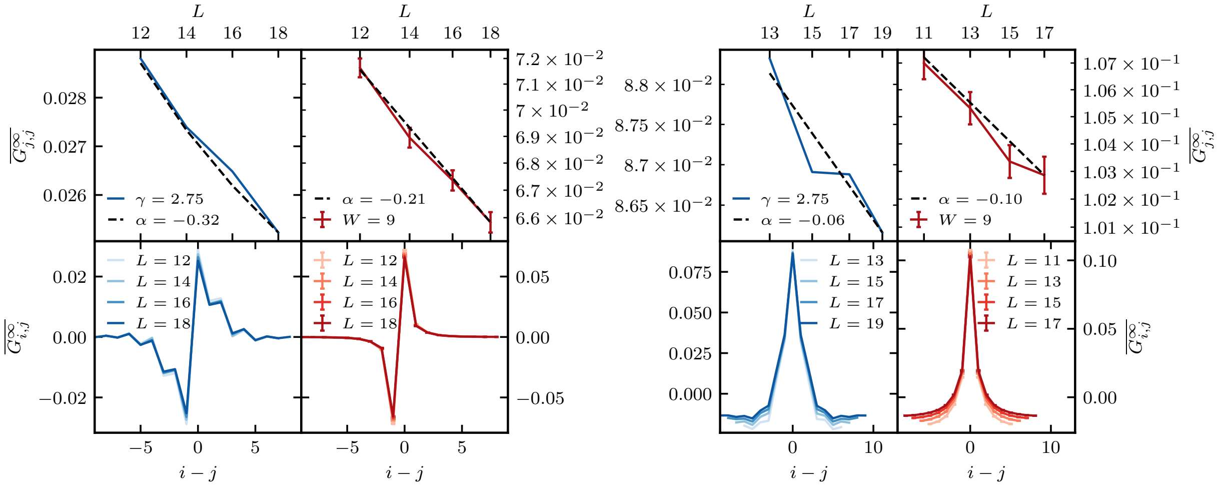

Supplementary Figure S1. **Excitation profile**. Infinite-temperature, infinite time average of connected spin-spin correlation function $\overline{G^{\infty}_{i,j}}$, for Stark-MBL system with $\gamma = 2.75$ (left panels) and symmetrized-MBL system with $W = 9$ (right panels) in the zero-magnetization sector. Top panels show autocorrelator for $j = \lceil L/2 \rceil$ as function of L on log-log scale, while bottom panels show the spatial profile for several system sizes (darker shades indicate larger systems).

Supplementary Note 4. SPATIAL PROFILE OF THE SPIN EXCITATION

The mean-square displacement (MSD) is lacking the spatial information on the spreading of the spin excitation, which is contained in the infinite-time averaged spin-spin correlation function,

$$\overline{G^{\infty}_{ij}} = \frac{1}{\mathcal{N}} \sum_{\alpha} \langle\alpha| \hat{S}^z_i |\alpha\rangle \langle\alpha| \hat{S}^z_j |\alpha\rangle . \tag{S34}$$

For delocalized systems with no memory of the initial condition this function is expected to vanish at all sites. In the bottom row of Supplementary Fig. S1 we calculate and plot $\overline{G^{\infty}_{ij}}$ for the Stark-MBL and symmetrized–MBL problem for a number of even (left) and odd (right) system sizes. For even system sizes the total magnetization is zero and the system is symmetric with respect to $\hat{P}$, which yields to the anti-symmetric shape $\overline{G^{\infty}_{ij}} = -\overline{G^{\infty}_{i\tilde{j}}}$. For odd system sizes the total magnetization is $1/2$ and the symmetry $\hat{P}$ is broken, such that the shape of the excitation $\overline{G^{\infty}_{ij}}$ doesn't have to be anti-symmetric. For even system sizes the correlation function is close to zero at all sites, except $i = j$ and $i = \tilde{j}$, indicating a relaxation to equilibrium, however for odd system sizes all sites appear to be away from zero.

The nonzero value of $\overline{G^{\infty}_{ij}}$ for $i = j$ and $i = \tilde{j}$, indicates some memory of the initial condition, however as the top row of Supplementary Fig. S1 shows this memory is decaying with the system size for both odd and even system sizes. We cannot reliably extract the dependence of the decay on the system size, but it is quite slow as one can learn from the qualitative power-law fits that are listed in the top row of Supplementary Fig. S1. It is important to note that non-vanishing of $\overline{G^{\infty}_{ij}}$ for a finite number of sites, is consistent with finite transport, since finite transport requires $\overline{\sigma^2_{\infty}} \sim L^2$ which includes a contribution from an extensive number of sites. Therefore existence of finite memory is not in contradiction to the proof in Supplementary Note 3. Here the memory of the excitation appears to fade away in the thermodynamic limit for both symmetry preserving and symmetry breaking systems.

Supplementary Note 5. EXCITATION SPREADING

In this section we consider the of the time-dependence of the excitation profile for both Stark-MBL and symmetrized–MBL systems for both symmetry preserving (even L) and symmetry breaking (odd L) system sizes. For this purpose we compute the positive MSD,

$$\sigma^2_{\text{sgn}}(t) = \sum_{i=1}^{L} (i-j)^2 \left| G^{\infty}_{ij}(t) - G^{\infty}_{ij}(0) \right| , \tag{S35}$$

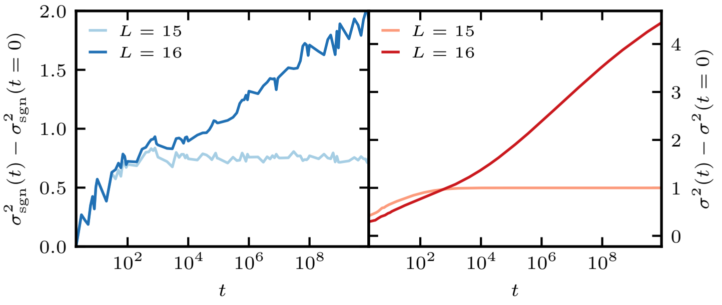

Supplementary Figure S2. **Spreading of the excitation with time**. Dynamics of the (positive) mean-squared displacement for Stark-MBL system with $\gamma = 2.75$ (left panel) and symmetrized-MBL system with $W = 9$ (right panel), plotted on semilog scale. The darker shade corresponds to zero-magnetization, while the lighter shade indicates results from a finite-magnetization sector. The standard deviation over disorder realizations for the symmetrized MBL is indicated by shaded areas.

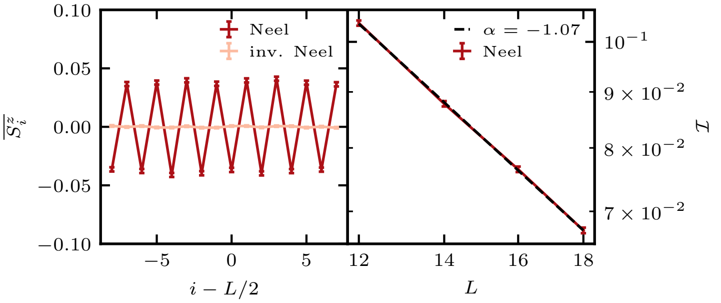

Supplementary Figure S3. ***Memory of initial condition at infinite time.*** *Left panel*: Infinite-time average of the magnetization profile starting from initial states related to the Néel state for the symmetrized-MBL system with $W = 9$ and L=16. Results are shown for the Néel state (dark red), $|\uparrow\downarrow\uparrow\downarrow\uparrow\downarrow\uparrow\downarrow\uparrow\downarrow\uparrow\downarrow\uparrow\downarrow\uparrow\downarrow\rangle$ and inverted Néel state, with the spins on the right half of the lattice flipped, $|\uparrow\downarrow\uparrow\downarrow\uparrow\downarrow\uparrow\downarrow\downarrow\uparrow\downarrow\uparrow\downarrow\uparrow\downarrow\uparrow\rangle$. *Right panel*: Asymptotic imbalance of the Néel state as a function of system size for even system sizes, plotted on a log-log scale.

which via triangle inequality bounds $\sigma^2_\infty(t) \leq \sigma^2_{\rm sgn}(t)$. We take $\sigma^2_{\rm sgn}(t)$, and not $\sigma^2_\infty(t)$, since for example for dipole preserving systems $\sigma^2_\infty(t) \leq C$ uniformly in time while there is still slow subdiffusive transport [1, 2].

From Supplementary Fig. S2 we see that while the dynamical behavior of Stark-MBL and symmetrized–MBL are very similar the long time behavior of symmetry preserving and symmetry breaking systems is very different. Symmetry preserving systems have an intermediate plateau after which the positive MSD grows logarithmically towards its infinite-time value. The height of the intermediate plateau decreases weakly with increasing the tilted-field or disorder strengths and delays the approach to the asymptotic plateau. The occurrence of quasi-degeneracies in the spectrum of symmetry preserving systems doesn't allow us to numerically compute the correct dynamics of the system beyond $t > 10^{16}$, we therefore don't present the dynamics beyond these time in Supplementary Fig. S2.

The dynamics of the positive MSD for symmetry breaking systems follows the dynamics of symmetry preserving systems up to time t_*, which increases with increasing the strength of the tilted field or the disorder, but does not depend on the system size. Interestingly, the intermediate plateau of symmetry preserving systems coincides with the asymptotic plateau of symmetry breaking systems. As explained in the main text the asymptotic value slowly increases with system size.

While here we present results of symmetry breaking by going to an even system size, we have observed very similar phenomenology if the symmetry is broken differently. For example, by considering a nonzero magnetization at even L or by adding weak disorder or curvature.

Supplementary Note 6. SYMMETRY BREAKING OF INITIAL CONDITIONS

The sensitivity of both Stark-MBL and symmetrized-MBL systems to symmetry breaking can also be observed via breaking the symmetry in the initial state and not the Hamiltonian. In the left panel of Supplementary Fig. S3 we

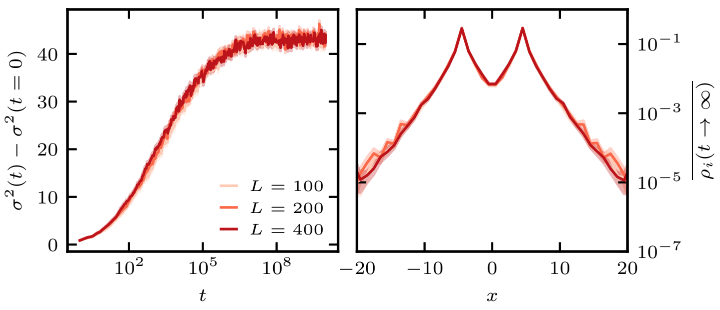

Supplementary Figure S4. **Spreading of a single electron**. The electron is initialized at site $x_0 = 5$, in a 1D lattice with mirror-symmetric on-site potential, $\mu_i = \mu_{-i} \in [-8, 8]$ and uniform nearest-neighbour hopping amplitude $t = 1$. The results are obtained from averaging over 1000 disorder realizations per system size. Left panel: Mean-squared displacement of the electronic density $\sigma^2(t) = \sum_{i=1}^{L} (i - x_0)^2 \rho_i(t)$ with as a function of time on semi-log scale. Right panel: Late-time average of the electronic density profile.

show the infinite-time average of $\left\langle \hat{S}_i^z(t) \right\rangle$,

$$\overline{S_i^z} = \overline{\left\langle \Psi \left| \hat{S}_i^z(t) \right| \Psi \right\rangle}, \tag{S36}$$

for the Néel state $|\text{Néel}\rangle = |\uparrow\downarrow\uparrow\downarrow\uparrow\downarrow\uparrow\downarrow\uparrow\downarrow\uparrow\downarrow\uparrow\downarrow\uparrow\downarrow\rangle$, which is even under $\hat{P}$. We see that $\overline{S_i^z}$ shows residual memory of this initial condition. We can quantify this memory using the imbalance,

$$\mathcal{I} = \sum_{i=1}^{L} (-1)^i \, \overline{S_i^z}, \tag{S37}$$

which slowly decays with the size of the system (see right panel of Supplementary Fig. S3). Flipping the spins in half of the system,

$$|\text{Inverted Néel}\rangle = \prod_{i=L/2+1}^{L} \sigma_i^x \, |\text{Néel}\rangle = |\uparrow\downarrow\uparrow\downarrow\uparrow\downarrow\uparrow\downarrow\downarrow\uparrow\downarrow\uparrow\downarrow\uparrow\downarrow\uparrow\rangle, \tag{S38}$$

results in a state which is neither odd nor even under $\hat{P}$. As can be seen from the left panel of Supplementary Fig. S3 any memory of the initial condition for this state is absent already for $L = 16$.

Supplementary Note 7. ANDERSON LOCALIZATION IN MIRROR-SYMMETRIC POTENTIAL

We provide numerical evidence that a system of noninteracting fermions with mirrorsymmetric but otherwise random on-site potential, $\mu_i = \mu_{-i}$, is localized in Supplementary Fig. S4. Note that the initial slow growth of the mean-squared displacement (MSD) is attributed to resonant tunneling of the fermion from its initial site (5 sites to the left of the center of the lattice) to the mirror-image of that site (see asymptotic fermionic density in right panel of Supplementary Fig. S4). Asymptotically, the MSD saturates at a value that is independent of the system size and only depends on the initial position of the excitation.

[1] Johannes Feldmeier, Pablo Sala, Giuseppe De Tomasi, Frank Pollmann, and Michael Knap, "Anomalous Diffusion in Dipole- and Higher-Moment-Conserving Systems," Phys. Rev. Lett. **125**, 245303 (2020).
[2] Guy Zisling, Dante M. Kennes, and Yevgeny Bar Lev, "Transport in Stark many-body localized systems," Phys. Rev. B **105**, L140201 (2022).
